# Practical recommendations for radium-223 treatment of metastatic castration-resistant prostate cancer

**DOI:** 10.1007/s00259-017-3756-7

**Published:** 2017-06-19

**Authors:** Yong Du, Ignasi Carrio, Giuseppe De Vincentis, Stefano Fanti, Harun Ilhan, Caroline Mommsen, Egbert Nitzsche, Francis Sundram, Wouter Vogel, Wim Oyen, Val Lewington

**Affiliations:** 1 Department of Nuclear Medicine & PET/CT, The Royal Marsden NHS Foundation Trust, Fulham Road, London, SW3 6JJ UK; 2 Hospital Sant Pau, Barcelona, Spain; 3 Policlinico Umberto I University Hospital Rome, Rome, Italy; 4 University Hospital Bologna, Bologna, Italy; 5 Ludwig-Maximilians-University Hospital, Munich, Germany; 6 Praxis für diagnostische und therapeutische Nuklearmedizin Berlin, Berlin, Germany; 7 Canton Hospital Aarau, Aarau, Switzerland; 8 University Hospital Southampton NHS Foundation Trust, Southampton, UK; 9 The Netherlands Cancer Institute, Amsterdam, Netherlands; 10 The Institute of Cancer Research, London, UK; 11 Guy’s and St Thomas’ NHS Foundation Trust, London, UK

**Keywords:** Bone metastases, Castration-resistant prostate cancer, Nuclear medicine, Radium dichloride, Ra-223, Targeted alpha therapy

## Abstract

**Purpose:**

Radium Ra 223 dichloride (radium-223, Xofigo®) is the first targeted alpha therapy for patients with castration-resistant prostate cancer and symptomatic bone metastases. Radium-223 provides a new treatment option for this setting, but also necessitates a new treatment management approach. We provide straightforward and practical recommendations for European nuclear medicine centres to optimize radium-223 service provision.

**Methods:**

An independent research consultancy agency observed radium-223 procedures and conducted interviews with all key staff members involved in radium-223 treatment delivery in 11 nuclear medicine centres across six countries (Germany, Italy, the Netherlands, Spain, Switzerland and the UK) experienced in administering radium-223. The findings were collated and discussed at a meeting of experts from these centres, during which key consensus recommendations were defined.

**Results:**

The recommendations cover centre organization and preparation; patient referral; radium-223 ordering, preparation and disposal; radium-223 treatment delivery/administration; and patient experience. Guidance includes structured coordination and communication within centres and multidisciplinary teams, focusing on sharing best practice to provide high-quality, patient-centred care throughout the treatment pathway.

**Conclusions:**

These expert recommendations are intended to complement existing management guidelines. Sharing best practice and experience will help nuclear medicine centres to optimize radium-223 service provision and improve patient care.

## Introduction

**Fig. 1:**
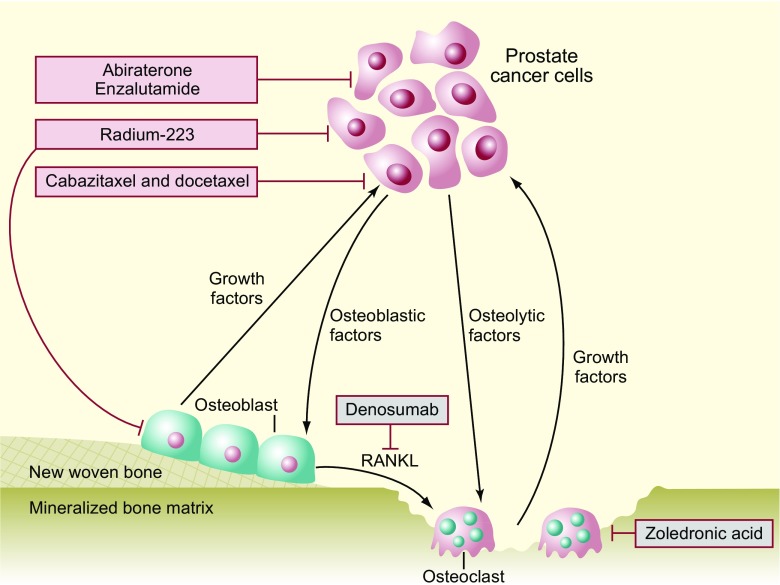
Dual mechanism of action of radium-223. RANKL, receptor activator of nuclear factor kappa-B ligand. Adapted by permission from Macmillan Publishers Ltd.: Nature Reviews Urology (Body et al. [15]), copyright 2015

Radium Ra 223 dichloride (radium-223, Xofigo®; Bayer AG, Germany) is a targeted alpha therapy approved for the treatment of castration-resistant prostate cancer (CRPC) with symptomatic skeletal metastases and no known visceral metastatic disease [1–3]. Targeted alpha therapy delivers alpha radiation to cancer cells and to the tumour microenvironment while minimizing toxicity to surrounding tissues (Fig. 1) [4–6]. Radium-223 acts as a calcium mimetic, binding to areas of increased bone turnover in skeletal metastases, subsequently inducing apoptotic effects through double-stranded DNA breaks [6, 7]. The short range of alpha particles emitted by radium-223 (∼100 μm, or ≤10 cell diameters) mitigates adverse effects on surrounding normal tissue [8]. Furthermore, radium-223 has a higher linear energy transfer than beta-emitting radionuclides, leading to more-targeted effects [8].

In the Phase 3 ALSYMPCA study among patients with CRPC and symptomatic bone metastases, radium-223 significantly improved overall survival (primary endpoint) and median time to first symptomatic skeletal-related event (both *p* < 0.001; Table 1), compared with placebo, and had a favourable safety profile [3]. An international early access Phase 3b study has provided further evidence of the radium-223 survival benefit in a cohort of patients with CRPC and bone metastases, who were considered representative of the typical metastatic CRPC population (Table 1) [9].

**Table 1:** Summary of key radium-223 efficacy data in mCRPC

Parameter	ALSYMPCA [3]			International EAP [9, 14]
	Radium-223 *n* = 614	Placebo *n* = 307	HR (95% CI)	*N* = 696
Median overall survival, months	14.9	11.3	0.70* (0.58–0.83)	16 (95% CI: 13–NE)
Median time to first SSE, months	15.6	9.8	0.66* (0.52–0.83)	18 (95% CI: 17–NE)
Median time to total ALP progression, months	7.4	3.8	0.17* (0.13–0.22)	8 (95% CI: NE–NE)
Median time to PSA progression, months	3.6	3.4	0.64* (0.54–0.77)	4 (95% CI: 3–4)

*p < 0.001

ALP, alkaline phosphatase; CI, confidence interval; EAP, early access programme; HR, hazard ratio; mCRPC, metastatic castration-resistant prostate cancer; NE, not estimated; PSA, prostate-specific antigen; SSE, symptomatic skeletal event

Radium-223 was introduced into clinical practice in 2013, and its benefits and recommendations for use are included in all major European and US treatment guidelines for CRPC [10–13]. The approved dose regimen of radium-223 is an activity of 55 kBq per kg body weight administered by six intravenous (IV) injections at 4-weekly intervals [1, 2]. Radium-223 is the first available targeted alpha therapy to significantly extend survival in CRPC. As such, its use in clinical practice is increasing. However, when offering radium-223 treatment, a new approach to service provision is necessary, owing to certain logistic and safety procedures. Furthermore, building collaboration between the different professional teams involved in managing patients with CRPC will require active input from nuclear medicine, as the main provider of radium-223.

We report the findings of a project to define expert recommendations from experienced European nuclear medicine centres to optimize the existing radium-223 service provision and guide new centres in establishing a radium-223 service. The focus is on practical aspects of radium-223 service delivery. Discussion of treatment sequencing with respect to other agents (e.g., enzalutamide or abiraterone) lies outside the scope of these recommendations.

## Material and methods

Eleven nuclear medicine experts from European centres across six countries (Germany, Italy, the Netherlands, Spain, Switzerland and the UK) experienced in using radium-223 were involved in a three-stage project to develop consensus recommendations. The initial stage involved several days of onsite observations of radium-223 procedures and patient treatment pathways in real-life clinical practice at each expert centre. This was carried out by an independent research consultancy agency (Alcimed, Paris, France). As part of this process, interviews were carried out with all key staff members, including nuclear medicine physicians, nurses, nuclear medicine technologists/radiographers, oncologists, radiopharmacists and physicists. The second stage involved debriefing workshops held in each expert centre with all key staff members. Best practices that had been observed both in general processes and in individual stages of the radium-223 patient treatment pathway (e.g., radium-223 injection) were evaluated. The workshops facilitated alignment between the staff members on best practice recommendations for that centre. The third stage was an expert meeting involving all authors of this manuscript to evaluate the findings and agree on the recommendations. The best practices identified from stages one and two were collated, evaluated and ranked by importance to identify key consensus best practice recommendations.

## Results

**Fig. 2:**
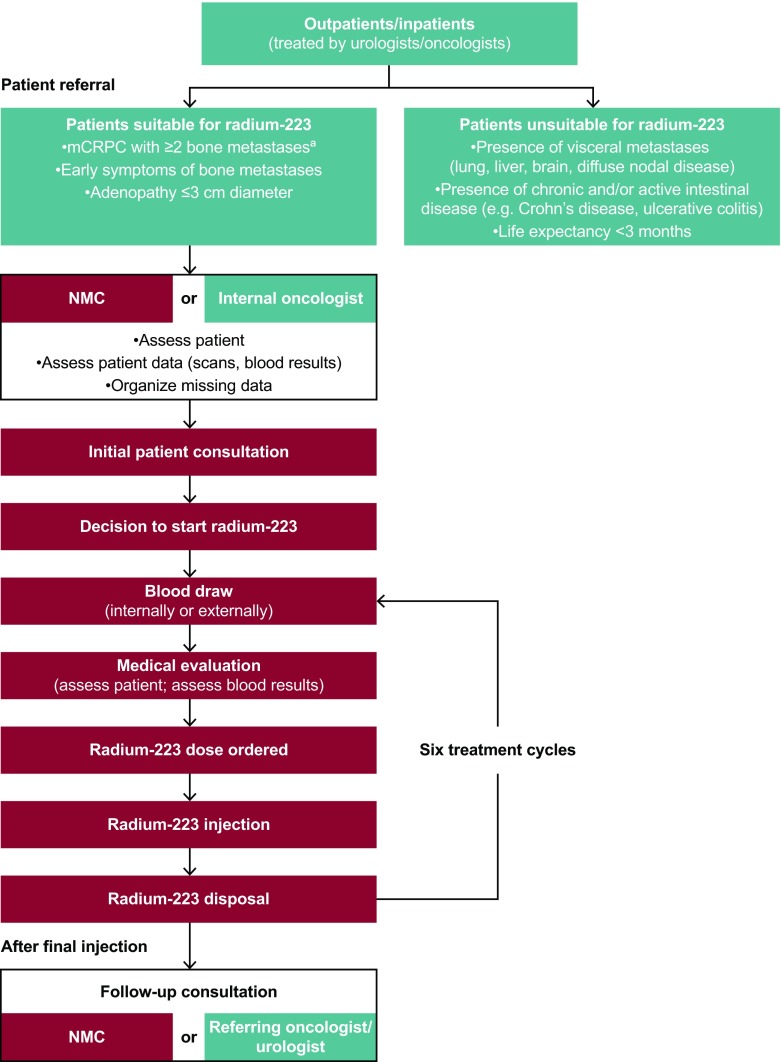
Radium-223 treatment pathway. ^a^Detectable with ^99m^Tc-phosphonate bone scan or ^18^F-NaF PET/CT scan. CT, computed tomography; F, fluoride; mCRPC, metastatic castration-resistant prostate cancer; NaF, sodium fluoride; NMC, nuclear medicine centre; PET, positron emission tomography; Tc, technetium

Consensus recommendations to optimize clinical practices throughout the radium-223 treatment pathway (Fig. 2) are described below, and the key points are summarized in Table 2. Guidance was grouped into five categories: centre organization and preparation; patient referral; radium-223 ordering, preparation and disposal; radium-223 treatment delivery/administration; and patient experience.

**Table 2:** Summary of key recommendations for radium-223 service provision

Category	Recommendation
Centre organization and preparation: structured coordination	Designate a key staff member to be the main radium-223 service coordinator to interact with everyone involved and oversee the complete treatment process Ensure close alignment of the nuclear medicine, oncology and urology services
Centre organization and preparation: staff training	Nuclear medicine physicians should be familiar with the management of patients with radium-223 and alternative treatment options; additional focused education may be required Provide practical training for new and existing staff members (nurses/technologists/scientific staff) with respect to preparing and administering radium-223 injections
Patient referral	Before the introduction of a radium-223 therapy service, provide background information about radium-223 for referring physicians, including a checklist to identify patients who are suitable for treatment
Radium-223 treatment delivery/administration: blood tests	Patients should have the option of either two short visits at the centre (one for blood test, one for injection) or one longer visit (blood test and injection on the same day) Alternatively, blood tests may be performed at the local GP surgery or local hospital. Provision of results by telephone can be used as an opportunity to discuss the patient’s health status
Radium-223 treatment delivery/administration: administration of radium-223	The nuclear medicine physician should undertake a consultation with the patient at each injection to help build a trusting relationship and ensure continuity of care
Patient experience: patient comfort and satisfaction	Patients should have one key worker throughout the treatment period
Patient experience: patient information	Discrepancies between information derived from different sources should be avoided wherever possible (e.g., provide a national unified radium-223 flyer)

GP, general practitioner

### Centre organization and preparation

#### Structured coordination

In most centres, the nuclear medicine physician is responsible for the therapy service provided for patients and referring physicians. The aim is to designate a key staff member to be the main radium-223 service coordinator to oversee the complete treatment pathway. This staff member will communicate with patients, referring physicians, nuclear medicine physicians and other nuclear medicine centre staff members, representatives from radiopharmacy (if present at the hospital), other departments and Bayer, the pharmaceutical company providing radium-223.

Close alignment of the nuclear medicine, oncology and urology services is recommended to facilitate a multidisciplinary approach to meet the complex needs of patients with prostate cancer and to ensure appropriate and efficient care for individuals receiving radium-223 treatment. This close interaction between departments facilitates identification of patients who are suitable for radium-223 treatment. Furthermore, it allows the evolution and establishment of a robust care pathway, ensuring prompt recognition of significant clinical changes during the 6-month course of therapy. As part of this multidisciplinary approach, the roles and responsibilities of the oncologist and nuclear medicine physician in providing care during the radium-223 treatment period need to be clarified. For example, the point of contact for the patient in general and in the event of a medical (oncological) emergency, both inside and outside of office hours, needs to be identified. Some centres are well supported by an acute oncology service (AOS) that provides 24-h advice, support and management for these patients.

Distribution of the main tasks associated with radium-223 treatment among nuclear medicine team members is advised to improve efficiency and reduce individual workload. Opportunities for nuclear medicine physicians to delegate tasks to other team members are encouraged. To ensure service continuity, introduction of an established system to provide cover for absent team members is recommended, particularly for the main radium-223 service coordinator, with trained deputies who can perform all necessary tasks. This will ensure a seamless service delivery, with no treatment delays. Dedicated days of the week for radium-223 consultations and treatments are recommended to assist department workflow. Depending on clinical demand, 1 defined treatment day each week would be feasible for most centres. However, to maintain flexibility for patients, centres may consider offering treatment on at least 2 days per week, where possible.

Availability of specific administrative documents and electronically available procedures for radium-223, together with detailed protocols for all radium-223-related processes, will facilitate treatment organization. These are typically developed by the centre, and address specific processes within the treatment pathway (e.g., radium-223 order forms and patient care plans that provide an overview of all six injections and summarize patient records during the treatment course). Detailed protocols and flow charts can help staff members to keep up to date with important processes (e.g., radium-223 disposal), and inform staff who provide cover for absent team members about tasks that they may perform infrequently.

#### Staff training

The treatment landscape for CRPC is evolving rapidly; therefore, it is recommended that nuclear medicine physicians are familiar with the management of patients with radium-223 and alternative treatment options; additional focused education may be required. In addition, practical onsite training with respect to preparing and administering radium-223 injections should be provided for new and existing staff members (nurses/technologists/scientific staff). For example, mentoring sessions could be arranged to ensure that new staff members undertake up to 20 injections under supervision before they dispense an injection by themselves. Staff rotation is helpful to ensure that every responsible staff member maintains their skills by preparing a radium-223 injection at least once every 2 weeks.

### Patient referral

Prior to the introduction of a radium-223 therapy service to a treatment centre, background information about radium-223 should be provided to referring physicians, including a checklist to identify patients who are suitable for the treatment. This will facilitate a streamlined and efficient referral pathway that minimizes any delays because of incomplete information. This will also ensure that patient expectations are managed, with appropriate patients who may benefit from radium-223 being referred, and patients who are unsuitable for treatment being offered alternative strategies.

Regular communication between referring physicians and the treating nuclear medicine physicians will establish solid working relationships and keep all parties informed of patients’ progress. This could be achieved via dedicated communication channels and face-to-face meetings. Such a multifaceted approach allows the referring physician to be updated about patients being treated in the nuclear medicine centre, and ensures that the nuclear medicine physician is informed of any general developments regarding the clinical status of the patient. Strong communication links are also recommended across the wider multidisciplinary and regional teams, in whatever format is most appropriate (e.g., regular multidisciplinary meetings to discuss individual patient management, including what to do if a patient becomes unwell during the treatment course, and regular service provision/audit meetings at a regional level). Regular multidisciplinary meeting attendance by the nuclear medicine physician (e.g., involving interdisciplinary experts committed to cancer care – such as ‘tumour boards’ – who discuss and agree upon specific treatment regimens for each patient) is also beneficial. This is important to reinforce continued awareness among other specialists about radium-223 as a therapeutic option for patients with CRPC. Furthermore, both referring physicians and nuclear medicine physicians can be informed of any updates or developments in nuclear medicine and in the prostate cancer management pathway.

### Radium-223 ordering, preparation and disposal

#### Radium-223 ordering

Many hospitals require dose-specific order numbers to be generated. To simplify the ordering process, order numbers for all six radium-223 injections can be created in advance to reduce administrative workload during the treatment phase. Minor, unforeseen, intercurrent events may lead to postponement of radium-223 therapy for a few days. When last-minute cancellations occur and postponement is not possible, it may be possible to switch orders to another patient of similar weight to avoid wasting the radium-223 dose.

#### Radium-223 preparation and disposal

It is recommended that dosage calculations be carried out and double-checked 1 day before scheduled treatment to allow efficient preparation of injections and, thus, save time on the day of treatment. Double-checking is important to ensure that the correct activity of radium-223 is administered. Ideally, only start the preparation of radium-223 for injection once patient attendance is confirmed to avoid unnecessary radiation exposure to staff members and wasting a radium-223 dose. After preparing the radium-223 syringe, a short plastic line filled with saline should be attached to the syringe tip as a safety precaution. This reduces the contamination risk in case of spillage.

All radioactive waste needs to be handled by properly trained staff and in accordance with local institutional regulations, but some countries (e.g., Germany) may require additional special procedures for the disposal of radium-223. Clear and visible separation of radium-223 waste helps to minimize exposure of staff members.

### Radium-223 treatment delivery/administration

#### Initial consultation

In the context of advancing underlying disease, it is important to start radium-223 treatment as soon as possible. No longer than 1 week between the referral and initial consultation by the nuclear medicine physician is recommended. The patient’s general practitioner (GP) should be notified that their patient is commencing radium-223 therapy in case any other medical intervention is required during treatment.

It is recommended that patients receive appointments for all six injections at treatment initiation. However, enough flexibility is needed for patients to reschedule appointments if they are unable to attend on a particular date. Notably, the survival benefit with radium-223 in the ALSYMPCA study was observed with some flexibility in the dosing schedule: the 4-weekly injections could be administered within a window of −3 days to +7 days, with a delay of no more than 4 weeks for recovery from adverse events [3]. Advance scheduling of all six injections has advantages for treatment centres and patients alike. Both can plan ahead, and the patient has a sense of security and can integrate the appointments into his daily life. Depending on local practice, written patient consent can also be obtained for all six injections in advance at the initial consultation.

It is important that patients receive detailed information about radium-223 from a reliable, well-informed source. Patients could receive their first detailed information on radium-223 from the referring physician so that they are not unduly deterred by information on, for example, side effects or radiation exposure. To achieve this aim, the referring physician needs to have previously received appropriate background information from the nuclear medicine department (see ‘Patient referral’ section). It is also recommended that additional background information on radium-223 therapy is sent to the patient prior to the first nuclear medicine consultation so that they can read it in advance.

It is helpful if a patient can meet both the nuclear medicine physician and key worker (i.e., key contact person) during the same visit (see ‘Patient experience’ section for further details of the key worker). This allows the key worker to be introduced to the patient as early as possible. Advanced planning of a structured sequence of topics for discussion ensures that all necessary information is covered during the initial consultation and sufficient time is dedicated to answering the patient’s questions.

#### Blood tests

It is recommended that patients receive reminders of their blood test appointments to streamline care. For patient convenience, the first blood test can be carried out on the same day as the initial consultation. This ensures that the blood test results are available quickly so the first injection can be scheduled shortly afterwards. In addition, results of the blood test may identify the need for, and allow arrangement of, interventions prior to treatment (e.g., blood transfusion for low haemoglobin level). For subsequent blood tests, patients should be able to choose either two short visits at the centre (one for blood test, one for injection) or one longer visit (blood test and radium-223 injection on the same day). Alternatively, blood tests may be performed closer to the patient’s home, at either their GP surgery or local hospital. In these instances, the nuclear medicine physician may be able to telephone the patient regarding his blood test results. These calls provide an opportunity to discuss the patient’s health status and confirm that administration of the next scheduled dose of radium-223 is still appropriate. This task could also be performed by the key worker or delegated to a radium-223 team member (e.g., experienced nurse or technician).

#### Administration of radium-223

It is recommended that the nuclear medicine physician undertakes a consultation with the patient when they attend for each injection. This helps to build a trusting relationship and ensures continuity of care. This is particularly important in centres where nuclear medicine physicians do not perform the radium-223 administration. When feasible, two staff members (not necessarily two doctors) should be present at each injection to ensure that the radioactive material is handled correctly. This approach also allows one staff member to prepare the injection while the other takes care of the patient. Radium-223 is delivered via an IV line and a three-way tap with Luer Lock connections. When regulations allow, and with the patient’s consent, an option is to permit attendance of relatives at the injection procedure.

Patients should receive written information after each treatment cycle to remind them of the hygiene precautions they should follow post-injection. Documentation confirming that the patient has received a radioactive injection and contact details for the centre should be provided.

#### Follow-up consultation

It is recommended that, where feasible, patients can choose to have their final consultation with the nuclear medicine or referring physician, or both. The optimal arrangement will vary according to local practice.

### Patient experience

#### Patient comfort and satisfaction

High-quality, patient-centred care is essential to ensure optimal patient outcomes. Patients should be supported by one key worker when they call or attend the centre throughout the treatment period to provide continuity. This arrangement provides patients with a familiar person to answer their questions and allows them to establish a relationship of trust from the outset. Often this role is carried out by a nurse or medical–technical radiological assistant (or related professional), but it could also be a nuclear medicine physician, the same person who acts as the main radium-223 service coordinator as described above, or another member of the nuclear medicine centre team. If the usual staff member is unavailable, it is recommended that patients be informed in advance that someone else will take care of them for the next appointment.

A key aim is that all appointments allow sufficient time to ensure patient comfort. About 60 min is suggested for the initial consultation to enable all relevant information to be reviewed and questions to be answered, and to obtain informed consent. However, it is recognized that this is a long appointment time that may not be feasible in all centres. Physicians should allow approximately 30 min for subsequent treatment visits in which the patient’s current health status can be assessed, the treatment room can be prepared and the injection administered. Allowing plenty of time ensures the patient does not feel rushed. It is recommended that the same dedicated treatment rooms are used at each visit so that patients feel relaxed in a familiar environment and know where they need to go for subsequent appointments. This can be particularly advantageous for anxious patients and helps to reduce the psychological stress associated with hospital attendances. Time delays should be avoided, and, in the context of patients with bone pain or restricted mobility, it helps to limit walking distances within the nuclear medicine centre. If the premises do not allow short walking distances, the centre should offer facilities to assist patients (e.g., wheelchair provision).

Systems may be implemented for patients to provide their feedback on their perception of the care that they have received. Examples include a questionnaire at the end of the treatment or feedback cards left in the waiting area, which can be completed informally and anonymously. Completed feedback cards can also be displayed in the waiting area by the hospital to demonstrate patient satisfaction and/or note areas for improvement.

#### Patient information

Written information and educational materials relating to radium-223 should be made available for patients and their carers. These can be given to the patient by the referring physician at, or prior to, the initial consultation and after individual treatment injections. These materials provide the patient with a reliable source of information at home, and may help to reduce the frequency of telephone calls to the centre. Examples include basic factsheets about radium-223 treatment or the experience of other patients (e.g., regarding the treatment, what to expect, etc.). Discrepancies between information derived from different sources should be avoided wherever possible. Several countries have developed a national unified radium-223 flyer that contains streamlined patient information and prevents potentially contradictory or confusing information being circulated.

Establishing a dedicated radium-223 telephone hotline allows patients to ask questions about their treatment. During usual business hours, this number would usually connect to the key worker in the nuclear medicine centre. An alternative number for use outside business hours (e.g., the AOS hotline) is also helpful. This information can be given to patients during their initial consultation.

## Discussion

Nuclear medicine now plays an increasingly important role in the management of patients with CRPC. Radium-223 is the first targeted alpha therapy with antitumour properties and proven significant survival benefits in men with CRPC and bone metastases. The establishment of radium-223 therapy into clinical practice requires active input from nuclear medicine specialists.

Here, we present a consensus view of best practice recommendations for centres that are currently treating patients with radium-223, and for centres that will soon initiate such treatment. It is important to note that differences in local institutional regulations and legal requirements exist. As a result, it is unlikely that every centre will be able to implement all recommendations, and, therefore, our intention has been to provide a comprehensive list of good practices that centres can implement, taking into account local regulations. This is not an exhaustive guide to radium-223 treatment provision and patient care, but is intended to complement country- or centre-specific management guidelines.

This work distils the combined knowledge and practical expertise of radium-223 service provision physicians from ten centres across six European countries. A robust scientific procedure was followed to identify key processes and recommendations from each centre and then analyse and validate the results. Although the recommendations provided here originate from European centres of excellence, it is likely that they are applicable to other healthcare systems outside of this geographical region, when used in combination with local regulations.

Our involvement in developing these best practice guidelines has been beneficial to our own nuclear medicine centres in improving processes. Sharing the key learnings from this process may help other nuclear medicine centres to facilitate the efficient use of radium-223 and improve patient care.
